# Four new species of the jumping spider genus *Portia* (Araneae, Salticidae) from China

**DOI:** 10.3897/zookeys.1068.72805

**Published:** 2021-11-03

**Authors:** Xin Xu, Xianjin Peng, Daiqin Li

**Affiliations:** 1 College of Life Sciences, Hunan Normal University, Changsha 410081, Hunan, China Hunan Normal University Changsha China; 2 Department of Biological Sciences, National University of Singapore, Singapore 117543, Singapore National University of Singapore Singapore Singapore

**Keywords:** Description, morphology, Hainan, Hong Kong, taxonomy

## Abstract

We diagnose and describe four new species of *Portia* Karsch, 1878 and describe for the first time the male of *P.zhaoi* Peng, Li & Chen, 2003 from China based on morphological characters. The females of *Portiabawang* sp. nov. have the narrowest epigyne orifice. The males of *Portiaerlangping* sp. nov. have the shortest embolus among all the species. The females of *Portiafajing* sp. nov. can be distinguished from other species by the anterior orifice margin, which is nearly parallel to the posterior orifice margin. The males of *Portiaxishan* sp. nov. can be identified by the tegular furrow which extends to form a membrane and by the tegular apophysis which is obscured; the females of *Portiaxishan* sp. nov. can be diagnosed by the slit-like epigynal orifice. The males of *P.zhaoi* have the longest embolus among all the species, and females can be diagnosed by the circular epigyne orifice and the longest copulatory ducts. To facilitate future identification, we also provide the GenBank accession codes of the DNA barcode gene, Cytochrome c oxidase subunit I (COI), for all the type specimens.

## Introduction

*Portia* Karsch, 1878 is the most thoroughly studied jumping spider genus and one of the best-known model systems for behavioural and evolutionary research in spiders (Su et al. 2007; Harland et al. 2012). Unlike typical jumping spiders, species of *Portia* are both cursorial predators and web builders. They build large, three-dimensional prey-catch webs (Jackson 1985). They also prey on other spiders by invading their webs and using aggressive mimicry to trick, then catch the resident spider. In addition, *Portia* species also eat insects ensnared in the alien web. Furthermore, all species of *Portia* show specialized prey-catching behaviour for a particular type of prey and have a preference for spiders as prey over insects (reviewed by Jackson and Pollard 1996; Li and Jackson 1996; Harland et al. 2012).

*Portia* was erected based on the female morphology of *Portiaschultzi* Karsch, 1878 (Karsch 1878). A taxonomic revision of the whole genus was completed by Wanless (1978). The monophyly of *Portia* is now strongly supported by both molecular and morphological data (Su et al. 2007; Maddison et al. 2014; Maddison 2015). *Portia* belongs to the subfamily Spartaeinae, tribe Spartaeini, subtribe Spartaeina (Maddison 2015). *Portia* is sister to *Cyrba* Simon, 1986 and *Paracyrba* Zabka & Kovac, 1996 (Su et al. 2007; Maddison 2014). To date, the genus contains 17 species worldwide, mainly distributed in the Oriental and Ethiopian regions, and specifically, 10 out of 17 *Portia* species occur in China (Wanless 1978; World Spider Catalog 2021). Peng and Li (2002) reported a taxonomic review of Chinese *Portia* species. The key to species of *Portia* was provided in the studies of Wanless (1978) and Peng and Li (2002) based on male and female genital morphology. Since Peng et al. (2003), only one new species of *Portia* has been reported from Taiwan (Zhang and Li 2005; World Spider Catalog 2021). In this study, after examining the vouchers collected in China, we identify and describe four new species of *Portia* and describe the male of *P.zhaoi* for the first time based on male and/or female genital morphology.

## Materials and methods

**Figure 1.**
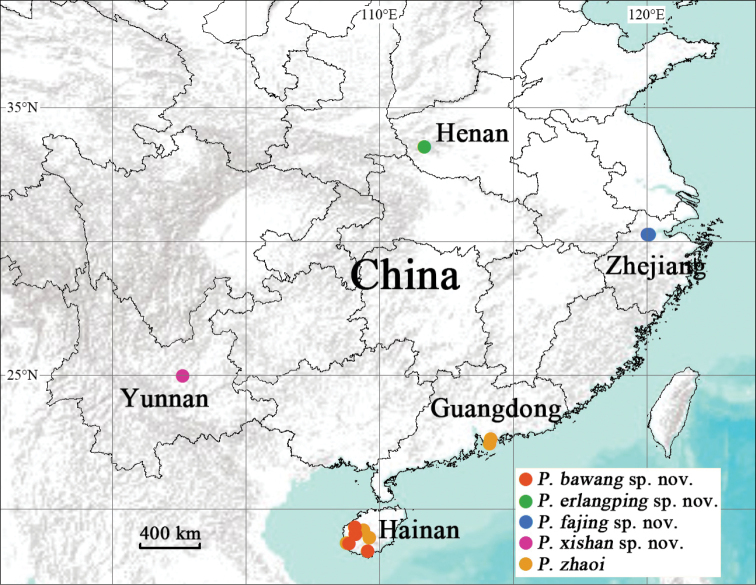
Map showing the collection sites of five *Portia* species in this study.

All specimens were collected from China (Fig. 1). We removed the right four legs of adults for molecular work, preserved them in 100% ethanol, and kept them at –80 °C. We preserved the remains of each specimen in 80% ethanol as a voucher for morphological examination. All voucher specimens are deposited at the College of Life Sciences, Hubei University, Wuhan, Hubei Province, China.

We examined and dissected the specimens under an Olympus SZ51 stereomicroscope. The soft tissues of female genitalia were degraded using 10 mg/ml trypsase (Bomei Biotech Company, Hefei, Anhui, China) for at least 3 h at room temperature. Male and female genitalia were photographed with a digital camera CCD mounted on an Olympus BX53 compound microscope, and then generated compound focused images with Helicon Focus v. 6.7.1. All measurements were made using a digital camera MC170HD mounted on a Leica M205C stereomicroscope and are given in millimeters. Leg and palp measurements are given in the following order: leg total length (femur + patella + tibia + metatarsus + tarsus), palp total length (femur + patella + tibia + tarsus).

Abbreviations used: AL = abdomen length; ALE = anterior lateral eyes; AME = anterior median eyes; AW = abdomen width; BL = body length; CF = cymbium flange; CL = carapace length; CW = carapace width; E = embolus; PLE = posterior lateral eyes; PME = posterior median eyes; T = tegulum; TA: tegular apophysis; TF = tegular furrow.

## Taxonomy

### Genus Portia

Taxon classificationAnimaliaAraneaeSalticidae

Karsch, 1878

147B82AA-6B13-5D0E-AFB4-CAEF5CEB1985

#### Type species.

*Portiaschultzi* Karsch, 1878

#### Diagnosis.

The genus *Portia* can be distinguished from other genera of the subfamily Spartaeinae by the dorsum of the abdomen with distinct tufts, the ventral tibiae with long fan-like fringes, and the malp palp with a dorsal cymbium flange (Zhang and Li 2005).

### Portia bawang sp. nov.

Taxon classificationAnimaliaAraneaeSalticidae

E2438FCB-2D3F-52D8-9540-077D2B974D86

http://zoobank.org/3833BB68-349D-4268-9D0B-F977EDB1DFA3

Figure 2

#### Type material.

***Holotype*:** China • 1 ♀; Hainan Province, Changjiang County, Bawang National Forest Park; 19.023°N, 109.103°E, alt. 692 m; 19 July 2012; F.X. Liu, D. Li and X. Xu leg.; DL-002-013-2012. ***Paratypes***: China • 1 ♀; same data as for the holotype; 19.027°N, 109.101°E, alt. 702 m; 7 August 2017; F.X. Liu, D. Li and X. Xu leg.; DL-003-002-2017 • 2 ♀♀; Hainan Province, Ledong County, Jianfeng National Forest Park; 19.296°N, 109.074°E, alt. 565–1005 m; 22 July 2012; F.X. Liu, D. Li and X. Xu leg.; DL-002-018-2012, DL-002-022-2012 • 1 ♀; Hainan Province, Lingshui County, Diaoluo National Forest Park; 18.400°N, 109.559°E, alt. 105 m; 21 June 2011; D. Li leg.; DL-005-006-2011.

#### Diagnosis.

**Figure 2.**
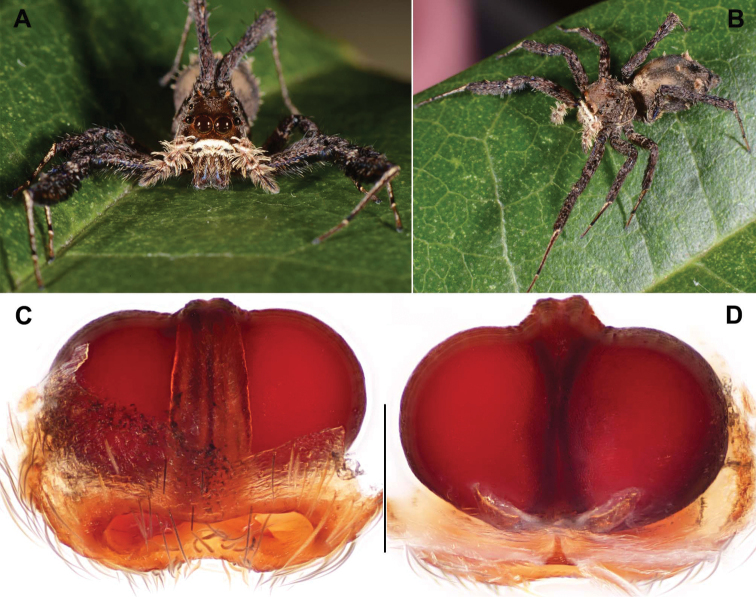
General somatic morphology and female genital anatomy of *Portiabawang* sp. nov. **A–D** DL-002-013-2012 (holotype) **A, B** female **C** epigyne, ventral view **D** vulva, dorsal view. Scale bar: 0.3 mm.

Females of *P.bawang* sp. nov. resemble those of *P.fimbriata*, *P.quei*, and *P.taiwanica* but can be distiguished from them by the epigyne orifice being narrowest (Fig. 2C; for comarison with known species, see fig. 8 in Wanless (1978), figs 664 and 666 in Peng et al. (1993), and fig. 4F in Zhang and Li (2005), respecitively); from those of *P.labiata* by the slightly straight anterior margin of posterior depression (Fig. 2C, D; see fig. 1C in Zhu et al. (2007)); from those of *P.heteroidea* by lacking a median septum (Fig. 2C; see figs 10–12 in Xie and Yin (1991)); from those of *P.fajing* sp. nov. and *P.xishan* sp. nov. by the epigyne orifice being narrowest and elliptical (Fig. 2C); from those of *P.zhaoi* by a smaller elliptical epigyne orifice and a shorter copulatory duct (Fig. 2C, D).

#### Description.

**Female** (holotype; Fig. 2A, B). Carapace greyish brown; ocular area yellow brown, with tufts of orange brown hairs around AME. Clypeus brown with dense ventral white hairs. Chelicerae dark brown with 3 small promarginal and 3 large retromarginal teeth. Maxillae and labium black-brown with reddish brown anterior margin. Sternum light brown, densely covered with creamy white hairs. Measurements: eye sizes: AME 0.80, ALE 0.31, PME 0.23, PLE 0.32, anterior eye row 2.46 wide, posterior eye row 2.30 wide, eye area 1.70 long; clypeus height 0.57; BL 8.17–9.46; holotype BL 9.46, CL 4.19, CW 3.09, AL 5.55, AW 3.68; palp 3.96 (1.27 + 0.58 + 0.76 + 1.35), leg I 12.56 (3.19 + 1.57 + 3.06 + 3.22 + 1.52), leg II 10.25 (2.95 + 1.41 + 2.26 + 2.41 + 1.22), leg III 8.48 (2.40 + 1.20 + 1.67 + 2.11 + 1.10), leg IV 13.89 (3.59 + 1.05 + 3.15 + 4.74 + 1.36). Leg formula 4123. Legs slender, ventral portion of tibiae fringed with long black hairs. Dorsum of abdomen black brown, anterior portion light brown with grey-white hairs, middle portion with a small patch and posterior portion with two oval patches, the three patches densely covered with greyish long hairs.

Female genitalia. Epigyne orifice undivided, highly sclerotised, elliptical, anterior orifice margin distinct and posterior margin wide, slightly curved; spermathecae large and spherical (Fig. 2C, D).

#### Etymology.

The species epithet, a noun in apposition, refers to the type locality.

#### Distribution.

Hainan (Changjiang, Ledong, Lingshui).

#### GenBank accession code of holotype.

OK235444.

### Portia erlangping sp. nov.

Taxon classificationAnimaliaAraneaeSalticidae

401795CE-DF36-53DC-90C2-3342119ADDA4

http://zoobank.org/8492FA45-A5CB-4000-95EB-6CD65D54C523

Figure 3

#### Type material.

***Holotype*:** China • 1 ♂; Henan Province, Nanyang City, Xixia County, Erlangping Town; 33.524°N, 111.688°E; 11 April 2013; F.X. Liu leg.; HN-013-001. ***Paratype***: China • 1 ♂; same data as for the holotype; HN-013-002.

#### Diagnosis.

**Figure 3.**
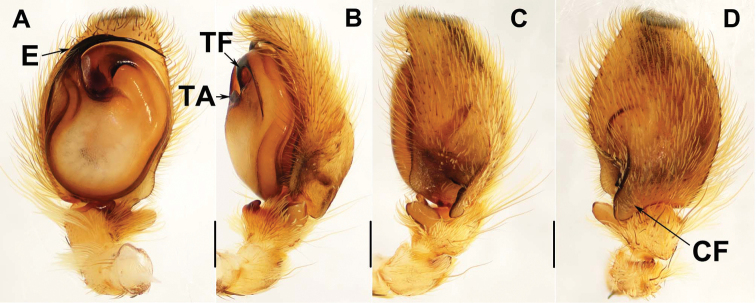
Male genital anatomy of *Portiaerlangping* sp. nov. **A–D** HN-013-001 (holotype) **A** palp, ventral view **B, C** palp, retrolateral view **D** palp, dorsal view. Scale bars: 0.3 mm.

Males of *P.erlangping* sp. nov. resemble those of *P.heteroidea* but can be distiguished from the latter by the tegulum having one curved furrow (Fig. 3A), while *P.heteroidea* has two furrows (see fig. 6 in Xie and Yin (1991)), and by the longer cymbium flange (Fig. 3D; see fig. 8 in Xie and Yin (1991)); from those of *P.albimana* by the longer embolus, larger cymbium flange and thicker retrolateral tibial apophysis (Fig. 3A, D; see fig. 12B–D in Wanless (1978)); from those of *P.assamensis*, *P.fimbriata*, *P.labiata*, *P.orientalis*, *P.quei*, *P.xishan* sp. nov., *P.taiwanica*, and *P.zhaoi* by the embolus being shortest (Fig. 3A; see figs 10D, 7C, and10A in Wanless (1978), fig. 6 in Murphy and Murphy 1983, fig. 661 in Peng et al. (1993), and fig. 4B in Zhang and Li (2005), respectively); in addition, from those of *P.assamensis* and *P.fimbriata* by the cymbium flange being thickest and longest (Fig. 3D; figs 10F, 7G in Wanless (1978), respectively); from those of *P.labiata* by the larger cymbium flange and thicker retrolateral tibial apophysis (Fig. 3D; see fig 10B in Wanless (1978)); from those of *P.orientalis*, *P.quei*, *P.taiwanica* and *P.zhaoi* by the bar-shaped retrolateral tibial apophysis (Fig. 3D; see fig. 6 in Murphy and Murphy 1983, and fig. 4D in Zhang and Li (2005), respectively).

#### Description.

**Male** (holotype). Carapace greyish brown with white band on thoracic groove and lateral margin. Ocular area yellow-brown, with tufts of yellow-brown hairs around AME. Clypeus black-brown without dense ventral white hairs. Chelicerae dark brown with 2 small promarginal and 3 large retromarginal teeth. Maxillae and labium black-brown with white anterior margin. Sternum black-brown, densely covered with creamy white hairs. Measurements: eye sizes: AME 0.64, ALE 0.24, PME 0.17, PLE 0.21, anterior eye row 1.78 wide, posterior eye row 1.65 wide, eye area 1.38 long; clypeus height 0.30; BL 5.84–6.45; holotype BL 5.84, CL 2.89, CW 2.14, AL 2.95, AW 1.60; leg I 8.40 (2.25 + 0.92 + 2.02 + 2.11 + 1.10), leg II 6.95 (1.90 + 0.94 + 1.57 + 1.60 + 0.94), leg III 6.46 (1.92 + 0.74 + 1.30 + 1.72 + 0.78), leg IV 9.45 (2.47 + 0.97 + 2.06 + 2.94 + 1.01). Leg formula 4123. Legs black-brown, slender, ventral portion of tibiae fringed with long black hairs. Dorsum of abdomen black-brown, anterior portion light brown with grey-white hairs, middle portion with a small triangular patch and posterior portion with two oval patches, the three patches densely covered with grey-white hairs.

Palp. Tibia with 3 apophyses, ventral one thick and short, intermediate one relatively slender, retrolateral one largest and bar-shaped in dorsal view (Fig. 3A–D). Embolus short and stout (Fig. 3A). Seminal duct clear and S-shaped. Tegulum with a deeply curved furrow and a membraneous apophysis (Fig. 3A, B). Cymbium flange robust, terminal portion overlapping on base of retrolateral tibial apophysis dorsally (Fig. 3D).

#### Etymology.

The species epithet, a noun in apposition, refers to the type locality.

#### Distribution.

Henan (Nanyang)

### Portia fajing sp. nov.

Taxon classificationAnimaliaAraneaeSalticidae

9047C3E2-1641-53DC-8968-4835CF1948E8

http://zoobank.org/E903C81D-1F7B-4966-91DD-93EB51627C4A

Figure 4

#### Type material.

***Holotype*:** China • 1 ♀; Zhejiang Province, Hangzhou City, Fajing Temple; 30.234°N, 120.095°E; alt. 79 m; 13 July 2013; F.X. Liu, D. Li, X. Xu and Z.T. Zhang leg.; DL-001-016-2013. ***Paratypes***: China • 4 ♀♀; same data as for the holotype, 30.228°N, 120.091°E; alt. 117 m; DL-001-015-2013, DL-002-016-2013, DL-003-016-2013, DL-003-017-2013.

#### Diagnosis.

**Figure 4.**
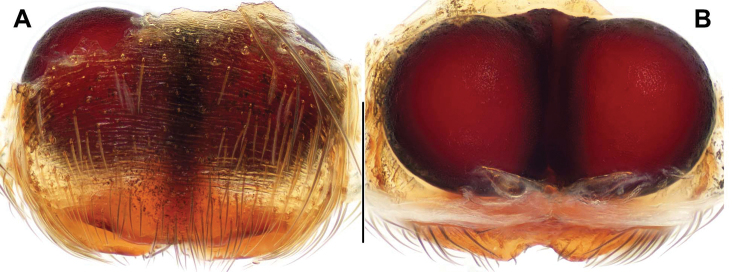
Female genital anatomy of *Portiafajing* sp. nov. **A, B** DL-001-016-2013 (holotype) **A** epigyne, ventral view **B** vulva dorsal view. Scale bar: 0.3 mm.

Females of *P.fajing* sp. nov. can be distinguished from those of *P.bawang* sp. nov., *P.labiata*, *P.quei*, and *P.taiwanica* by the slit-like epigynal orifice, the anterior orifice margin nearly parallel to the posterior orifice margin ventrally, and the W-shaped posterior epigynal margin (Fig. 4A, B; see fig. 1C in Zhu et al. (2007), fig. 664 and 665 in Peng et al. (1993), and fig. 4F in Zhang and Li (2005), respecitively); from those of *P.xishan* sp. nov. by the W-shaped posterior orifice margin (Fig. 4B); from those of *P.fimbriata* and *P.zhaoi* by the copulatory duct being shortest (Fig. 4B; see fig. 8D, E in Wanless (1978))

#### Description.

**Female** (holotype). Carapace brown; ocular area yellow-brown, with tufts of orange-brown hairs around AME. Clypeus brown with dense ventral white hairs. Chelicerae dark brown with 3 small promarginal and 3 large retromarginal teeth. Maxillae and labium reddish brown with yellow-brown anterior margin. Sternum yellow-brown, densely covered with creamy white hairs. Measurements: eye sizes: AME 0.68, ALE 0.30, PME 0.21, PLE 0.29, anterior eye row 2.01 wide, posterior eye row 1.87 wide, eye area 1.55 long; clypeus height 0.35; BL 6.56–7.64; holotype BL 7.64, CL 3.23, CW 2.58, AL 4.71, AW 3.22; palp 2.80 (0.77 + 0.48 + 0.59 + 0.96), leg I 8.74 (2.37 + 1.10 + 2.01 + 2.23 + 1.03), leg II 7.17 (2.01 + 1.06 + 1.60 + 1.61 + 0.89), leg III 6.61 (1.83 + 0.90 + 1.35 + 1.74 + 0.79), leg IV 10.84 (2.76 + 1.12 + 2.43 + 3.62 + 0.91). Leg formula 4123. Legs brown, the ventral portion of tibiae fringed with long black hairs. Dorsum of abdomen greyish brown, posterior portion with two circular patches densely covered with grey-white long hairs.

Female genitalia. Epigyne orifice undivided, highly sclerotised, transverse, slit-like, posterior orifice margin W-shaped; spermatheca large and spherical (Fig. 4B).

#### Etymology.

The species epithet, a noun in apposition, refers to the type locality.

#### Distribution.

Zhejiang (Hangzhou).

#### GenBank accession code of holotype.

OK235443.

### Portia xishan sp. nov.

Taxon classificationAnimaliaAraneaeSalticidae

92230B49-D337-517E-ACF5-5B2996ADCBF7

http://zoobank.org/8B8AD618-B4D9-4128-82D2-033436119365

Figure 5

#### Type material.

***Holotype*:** China • 1 ♂; Yunnan Province, Kunming City, Western Mountains; 24.962°N, 102.631°E, alt. 2172 m; 16 August 2006; F.X. Liu and Q.Q. Liu leg.; LQ-18-06. ***Paratypes***: China • 1 ♂ 3 ♀♀; same data as for the holotype; LQ-18-06A/06B/06C/06D • 8 ♀♀; same data as for the holotype; 11 November 2020; L. Yu and X.R. Miao leg.; P2020001, P2020010, P2020011, P2020034, P2020039, P2020053, P2020054, P2020055.

#### Diagnosis.

**Figure 5.**
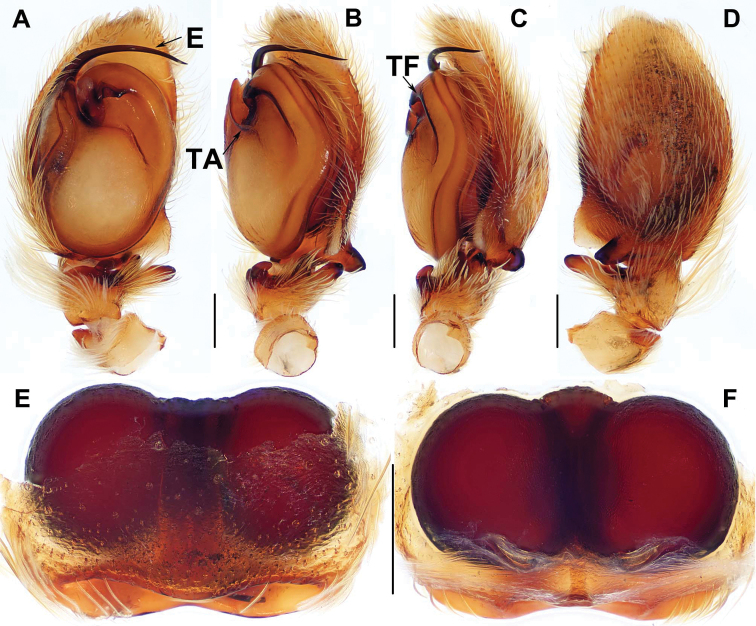
Male and female genital anatomy of *Portiaxishan* sp. nov. **A–D** LQ-18-06 (holotype) **A** palp, ventral view **B, C** palp, retrolateral view **D** palp, dorsal view **E, F** LQ-18-06B **E** epigyne, ventral view **F** vulva, dorsal view. Scale bars: 0.3 mm.

Males of *P.xishan* sp. nov. can be distinguished from those of *P.albimana* by the longer embolus, larger cymbium flange and thicker, bar-shaped retrolateral tibial apophysis (Fig. 5A–D; see fig. 12B–D in Wanless (1978)); from those of *P.assamensis*, *P.erlangping* sp. nov., *P.fimbriata*, *P.labiata*, *P.orientalis*, *P.quei*, *P.taiwanica*, and *P.zhaoi* by the tegular furrow extending a membranous apophysis ventrally, the tegular apophysis obscure, and the embolus basally with a spinule (Fig. 5A–C; see figs 10D, 7C, and 10A in Wanless (1978), fig. 6 in Murphy and Murphy 1983, fig. 661 in Peng et al. (1993), and fig. 4B in Zhang and Li (2005), respectively); in addition, from those of *P.assamensis* and *P.fimbriata* by the embolus being shortest (Fig. 5A–C; see figs 10D and 7C in Wanless (1978)); from those of *P.erlangping* sp. nov. by the embolus being longer (Fig. 5A); from those of *P.labiata* by the shorter cymbium flange and thicker bar-shaped retrolateral tibial apophysis (Fig. 4D; see fig. 10B in Wanless (1978)); from those of *P.orientalis*, *P.quei*, *P.taiwanica*, and *P.zhaoi* by the longer embolus and bar-shaped retrolateral tibial apophysis (Fig. 5A–D; see fig. 6 in Murphy and Murphy 1983, fig. 661 in Peng et al. (1993), and fig. 4B in Zhang and Li (2005), respectively). Females of *P.xishan* sp. nov. differ from those of *P.bawang* sp. nov. by having a slightly wider epigynal orifice (Fig. 5E); from those of *P.fajing* sp. nov. by the larger epigyne orifice and distinctly sclerotised anterior orifice margin (Fig. 5E); from those of *P.labiata*, *P.quei*, and *P.taiwanica* by the slit-like epigynal orifice (Fig. 5E; see fig. 1C in Zhu et al. (2007), fig. 664 and 665 in Peng et al. (1993), and fig. 4F in Zhang and Li (2005), respecitively); from those of *P.fimbriata* and *P.zhaoi* by the copulatory duct being shortest (Fig. 5F; see fig. 8D, E in Wanless (1978)).

#### Description.

**Male** (Holotype). Carapace black-brown with white band on thoracic groove and lateral margin. Ocular area yellow-brown, with tufts of yellow-brown hairs around AME. Clypeus black-brown without dense ventral white hairs. Chelicerae dark brown with 5 small promarginal and 2 large retromarginal teeth. Maxillae and labium black-brown with yellow-brown anterior margin. Sternum yellow-brown, densely covered with creamy white hairs. Measurements: eye sizes: AME 0.59, ALE 0.30, PME 0.19, PLE 0.28, anterior eye row 1.78 wide, posterior eye row 1.69 wide, eye area 1.32 long; clypeus height 0.42; BL 5.72–6.21; holotype BL 5.72, CL 2.87, CW 2.17, AL 2.75, AW 1.54; leg I 8.02 (2.04 + 0.91 + 1.81 + 2.04 + 1.22), leg II 6.80 (1.95 + 0.81 + 1.38 + 1.71 + 0.95), leg III 6.58 (1.87 + 0.86 + 1.29 + 1.64 + 0.92), leg IV 9.85 (2.61 + 0.92 + 2.09 + 3.17 + 1.06). Leg formula 4123. Legs black-brown, slender, the ventral portion of tibiae fringed with long black hairs. Dorsum of abdomen greyish brown, anterior portion light brown with grey-white hairs, three pairs of oval patches densely covered with grey-white hairs, the posterior pair largest.

Palp. Tibia with 3 apophyses, ventral one thick and short, intermediate one relatively slender, retrolateral one largest and bar-shaped in dorsal view (Fig. 5A–D). Embolus short and stout, with a spinule basally in retrolateral view (Fig. 5A–C). Seminal duct clear and S-shaped. Tegulum with a curved furrow extending a membranous apophysis ventrally and an obscure tegular apophysis (Fig. 5A–C). Cymbium flange robust, terminal portion close to middle portion of retrolateral tibial apophysis dorsally (Fig. 5D).

**Female (LQ-18-06B).** Carapace yellow-brown; ocular area yellow-brown, with tufts of greyish brown hairs around AME. Clypeus brown with densely ventral white hairs. Chelicerae dark brown with 4 small promarginal and 3 large retromarginal teeth. Maxillae and labium black-brown with yellow brown to white hairs on anterior margin. Sternum brown, densely covered with creamy white hairs. Measurements: eye sizes: AME 0.67, ALE 0.30, PME 0.23, PLE 0.27, anterior eye row 1.93 wide, posterior eye row 1.86 wide, eye area 1.67 long; Clypeus height 0.42; BL 5.83–7.66; LQ-18-06B: BL 6.10, CL 2.79, CW 2.44, AL 3.42, AW 2.11; palp 2.93 (0.95 + 0.31 + 0.58 + 1.09), leg I 7.20 (2.09 + 0.88 + 1.70 + 1.52 + 1.01), leg II 6.17 (2.01 + 0.95 + 1.15 + 1.15 + 0.91), leg III 6.02 (1.82 + 0.72 + 1.19 + 1.39 + 0.90), leg IV 7.72 (2.85 + 0.78 + 1.46 + 1.71 + 0.92). Leg formula 4123. Legs brown, ventral portion of tibiae fringed with long black hairs. Dorsum of abdomen brown, anterior margin with numerous long white hairs, posterior portion with two circular patches densely covered with grey-white hairs.

Female genitalia. Epigyne orifice undivided, highly sclerotised, transverse, spindly, posterior orifice margin slightly curved; spermathecae large and spherical (Fig. 5E, F).

#### Etymology.

The species epithet, a noun in apposition, “xishan” means Western Mountains in Chinese and refers to the type locality.

#### Distribution.

Yunnan (Kunming).

#### GenBank accession code of holotype.

OK235446.

### Portia zhaoi

Taxon classificationAnimaliaAraneaeSalticidae

Peng, Li & Chen, 2003

F35A91BB-1BE3-53B2-88D0-6D28C2CA9E08

Figure 6

Portia zhaoi Peng, Li & Chen, 2003: 50, figs 1–4; Peng 2020: 356, fig. 255a–d.

#### Type material examined.

***Holotype*:** China • 1 ♀; Guangxi Zhuang Autonomous region, Dongxing County, Rongguang Tea Plantation; 21.29°N, 108.02°E; 13 August 1992; F.X. Liu leg.

#### Additional material examined.

China • 1 ♂ 1 ♀; Hainan Province, Wuzhishan City, Shuiman Town, Yongxun Village; 18.903°N, 109.623°E, alt. 551 m; 25 July 2012; F.X. Liu, D. Li and X. Xu leg.; DL-002-024-2012, DL-001-024-2012; 1 ♂; Shenzhen, Xianhu Lake; 22.583°N, 114.169°E, alt. 66 m; 14 June 2012; F.X. Liu, D. Li and X. Xu leg.; DL-007-2012; • 1 ♀; Shenzhen, Yinhu Lake; 22.58°N, 114.08°E; 15 June 2012; F.X. Liu, D. Li and X. Xu leg.; DL-008-2012 • 1 ♂ 2 ♀♀; Hainan Province, Changjiang County, Bawang National Forest Park; 19.027°N, 109.101°E, alt. 702 m; 7 August 2017; F.X. Liu, D. Li and X. Xu leg.; LID-001-002-2017, LID-002-002-2017, LID-004-002-2017 • 1 ♀; Hong Kong, Kadoorie Farm and Botanic Garden; 22.424°N, 114.125°E, alt. 571 m; 12 April 2012; F.X. Liu, D. Li and X. Xu leg.; DL-001-2012 • 1 ♂; Hainan Province, Ledong County, Jianfeng Town, Institute of Tropical Forestry; 18.703°N, 108.789°E, alt. 129 m; 21 July 2012; F.X. Liu, D. Li and X. Xu leg.; DL-004-017-2012 • 1 ♂; Hainan Province, Yacha Town, 1^st^ Burei Village; 19.193°N, 109.418°E, alt. 268 m; 18 July 2012; F.X. Liu, D. Li and X. Xu leg.; DL-011-2012.

#### Diagnosis.

**Figure 6.**
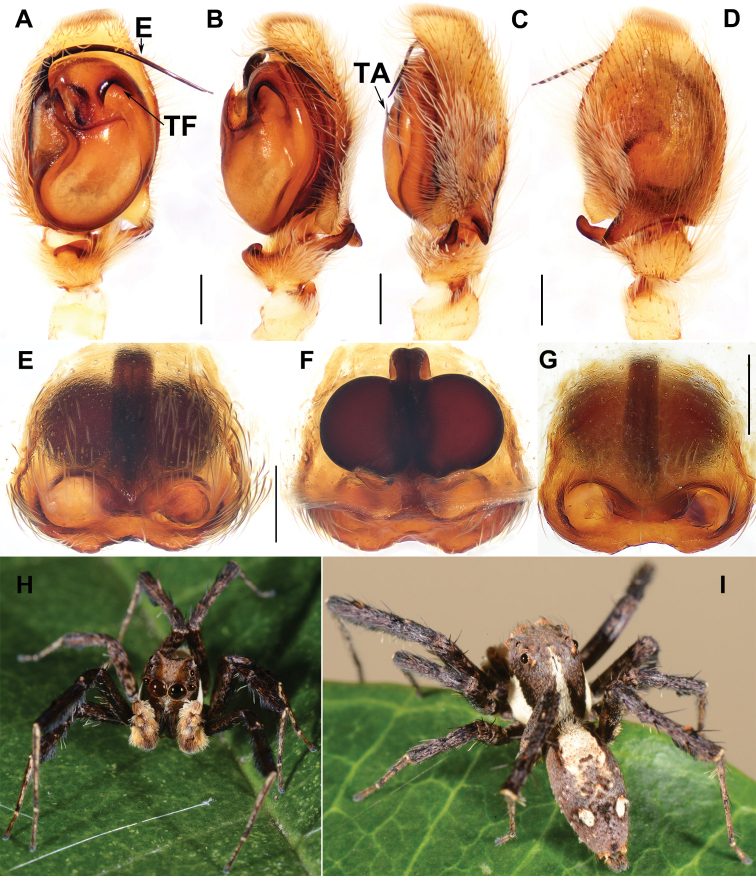
Male and female genital anatomy of *Portiazhaoi* Peng, Li & Chen, 2003 **A–D** DL-002-024-2012 **A** palp, ventral view **B, C** palp, retrolateral view **D** palp, dorsal view **E, F** DL-001-024-2012 **G** holotype deposited in the Institute of Zoology, Chinese Academy of Sciences, Beijing **E, G** epigyne, ventral view **F** vulva dorsal view **H, I** male. Scale bars: 0.3 mm.

Males of *P.zhaoi* can be distinguished from those of all other *Portia* species by having the longest embolus (Fig. 6A); in addition, from those of *P.erlangping* sp. nov., *P.fimbriata* and *P.xishan* sp. nov. By the finger-shaped retrolateral tibial apophysis (Fig. 6A, B, D; see 7G in Wanless (1978)); from those of *P.quei* by the thicker ventral tibial apophysis (Fig. 6B; see fig. 661 in Peng et al. (1993)). Females of *P.zhaoi* can be distinguished from those of *P.labiata* by its longer copulatory ducts (Fig. 6E, F; see fig. 1D in Zhu et al. (2007)); from those of *P.bawang* sp. nov., *P.fajing* sp. nov., *P.fimbriata*, *P.quei*, *P.taiwanica*, and *P.xishan* sp. nov. by the circular epigyne orifice (Fig. 6E; see fig. 8C–E in Wanless (1978), fig. 664 and 666 in Peng et al. (1993), and fig. 4F in Zhang and Li (2005), respecitively).

#### Description.

**Male** (**DL-002-024-2012**, Fig. 6G, H). Carapace yellow-brown with white band on thoracic groove and lateral margin. Ocular area yellow-brown, with tufts of yellow hairs around AME. Clypeus black-brown without dense ventral white hairs. Chelicerae dark brown with 4 small promarginal and 3 large retromarginal teeth. Maxillae and labium black-brown with yellow-brown anterior margin. Sternum yellow-brown, densely covered with creamy white hairs. Measurements: eye sizes: AME 0.68, ALE 0.29, PME 0.17, PLE 0.32, anterior eye row 1.88 wide, posterior eye row 1.67 wide, eye area 1.50 long; clypeus height 0.32; BL 5.62–7.63; holotype BL 5.69, CL 2.97, CW 2.32, AL 2.84, AW 1.32; leg I 9.24 (2.52 + 0.69 + 2.31 + 2.56 + 1.16), leg II 7.58 (2.26 + 0.86 + 1.61 + 2.02 + 0.83), leg III 6.54 (1.90 + 0.88 + 1.29 + 1.70 + 0.77), leg IV 9.54 (2.89 + 0.85 + 1.91 + 2.87 + 1.02). Leg formula 4123. Legs black brown, slender, the ventral portion of tibiae fringed with long black hairs. Dorsum of abdomen brown, anterior portion light brown with grey-white hairs, one pair of oval patches covered with dense grey-white hairs.

Palp. Tibia with 3 apophyses, ventral one thick and short, intermediate one relatively slender, retrolateral one largest and finger-shaped in dorsal view (Fig. 6A–D). Embolus slender and long (Fig. 6A). Seminal duct clear and S-shaped. Tegulum with a deeply curved furrow, and a triangular membraneous apophysis (Fig. 6A–C). Cymbium flange robust, triangular, terminal portion overlapping on middle portion of retrolateral tibial apophysis dorsally (Fig. 6D).

#### Redescription.

**Female (DL-001-024-2012).** Carapace black brown; ocular area yellow brown, with tufts of greyish hairs around AME. Clypeus brown with densely ventral white hairs. Chelicerae dark brown with 3 small promarginal and 3 large retromarginal teeth. Maxillae and labium black-brown with yellow brown anterior margin. Sternum yellow-brown, densely covered with creamy white hairs. Measurements: eye sizes: AME 0.79, ALE 0.35, PME 0.26, PLE 0.37, anterior eye row 2.29 wide, posterior eye row 2.13 wide, eye area 1.81 long; clypeus height 0.42; BL 7.18–8.24; DL-001-024-2012: BL 7.85, CL 4.05, CW 3.07, AL 4.17, AW 2.24; palp 3.64 (0.80 + 0.64 + 0.75 + 1.45), leg I 11.32 (3.16 + 1.39 + 2.64 + 2.92 + 1.21), leg II 9.52 (2.81 + 1.36 + 2.13 + 2.33 + 0.89), leg III 8.20 (2.39 + 1.05 + 1.67 + 2.23 + 0.86), leg IV 13.98 (3.71 + 1.44 + 2.98 + 4.94 + 0.91). Leg formula 4123. Legs black-brown, the ventral portion of tibiae fringed with long black hairs. Dorsum of abdomen brown, anterior margin with numerous long white hairs, posterior portion with three oval patches densely covered with grey-white hairs, the middle one small and the posterior two large.

Female genitalia. Epigyne orifice undivided, highly sclerotised, circular, anterior orifice margin distinct, posterior orifice margin slightly curved; spermathecae large and spherical (Fig. 6E, F).

#### Etymology.

The species epithet, a noun in apposition, refers to the type locality.

#### Distribution.

Guangxi, Hainan, Hong Kong, Shenzhen.

#### GenBank accession code of DL-002-024-2012.

OK235445.

## Supplementary Material

XML Treatment for Portia

XML Treatment for Portia bawang

XML Treatment for Portia erlangping

XML Treatment for Portia fajing

XML Treatment for Portia xishan

XML Treatment for Portia zhaoi
